# Genetic insights into congenital and infantile nephrotic syndrome: predicting severity and informing care

**DOI:** 10.1007/s00467-026-07407-2

**Published:** 2026-06-26

**Authors:** Ewelina D. Hejenkowska, Paul Kruszka, Agnieszka Swiatecka-Urban

**Affiliations:** 1 Department of Pediatrics, Division of Nephrology, University of Virginia School of Medicine, Charlottesville, VA 22903 USA; 2 Department of Pediatrics, Division of Genetics, University of Virginia School of Medicine, Charlottesville, VA 22903 USA

**Keywords:** Congenital nephrotic syndrome, Infantile nephrotic syndrome, Genetic testing, Next-generation sequencing

## Abstract

Nephrotic syndrome (NS) is the most common chronic glomerular disorder in pediatric populations, yet its manifestation during the neonatal or infantile period often portends a severe, treatment-resistant monogenic form. While recent studies reveal marked heterogeneity in the clinical course and severity of congenital and infantile NS, critical gaps remain in understanding its underlying mechanisms. This review synthesizes current evidence on the genetic landscape of the early-onset NS, highlighting how advances in next-generation sequencing and molecular diagnostics have refined genotype–phenotype correlations. We discuss how these insights are reshaping disease classification, enabling early prognostic stratification, and informing personalized management strategies. Emphasis is placed on the transformative role of genetic testing in guiding clinical care and uncovering novel therapeutic avenues for this complex pediatric kidney disorder.

**Graphical abstract:**

**Figure:**
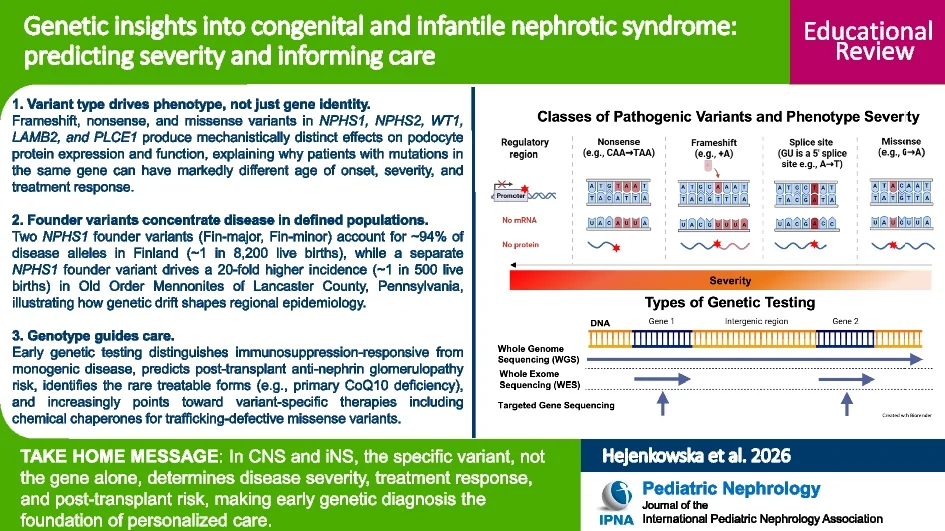
A higher-resolution version of the Graphical abstract is available as Supplementary information

**Supplementary Information:**

The online version contains supplementary material available at https://doi.org/10.1007/s00467-026-07407-2.

## Introduction

Nephrotic syndrome (NS) is the most common chronic glomerular disease in children and presents with heavy proteinuria, hypoalbuminemia, edema, and dyslipidemia [1–3]. When NS manifests during the first year of life, it is most often caused by monogenic defects and is associated with severe symptoms and resistance to immunosuppressive therapy [4–6]. Early-onset NS is further classified into congenital NS (CNS), presenting within the first three months of life, and infantile NS (iNS), presenting between 4–12 months of age [1]. CNS is most frequent in Finland, with the incidence of approximately 12 per 100,000 newborns compared to 1–3 per 100,000 live births worldwide. CNS and iNS are clinically severe, frequently present with extrarenal complications, and may progress to kidney failure. Both forms result from variants in genes encoding proteins critical for the glomerular filtration barrier structure and function, including *NPHS1*, *NPHS2*, *WT1*, *PLCE1*, and *LAMB2* [7–10]*.* Variants in *NPHS1,* encoding nephrin and *NPHS2,* encoding podocin, are the most common causes of CNS [8, 10–12]. In the Finnish population, two *NPHS1* variants, Fin-major and Fin-minor, account for approximately 94% of mutated alleles, with *NPHS1* overall responsible for 98% of CNS cases [7, 13]. In non-Finnish populations, *NPHS2* is numerically more frequent than *NPHS1* across the full congenital period (0–3 months) (39.1% vs. 22.5%) [8, 11, 12, 14]. However, when onset is stratified by age, *NPHS1* variants are the leading cause of CNS presenting within the first week of life, while *NPHS2* variants are the most common cause of CNS presenting after the first month of life and of iNS overall [8, 14, 15]. Variants in *LAMB2*, encoding laminin subunit β−2, account for 4.4% and *WT1*, encoding the Wilms tumor 1 protein account for 2.2% of CNS cases [11, 14]. *COQ2, COQ6, PDSS2, SGPL1, TRPC6, INF2,* and *ACTN4* variants are usually associated with *iNS* [9, 16–23]. The clinical severity and prognosis are influenced by the genetic defect and the extent of extrarenal manifestations.

Despite advances in supportive care, morbidity and mortality of CNS and iNS remain high [8, 17, 24–27]. The 2020 expert opinion paper and 2021 Consensus Statement from the European Reference Network for Kidney Diseases (ERKNet) and the European Society for Pediatric Nephrology (ESPN) management of CNS recommend genetic screening as the first-line diagnostic measure for all patients with this condition [28, 29]. Beyond age of onset, additional clinical clues may point to the underlying genetic defect. The presence of placental enlargement, prematurity and low birth weight typically points to *NPHS1* null variants. Prenatal or neonatal chronic kidney disease at diagnosis, which occurs more commonly with *WT1*, *PLCE1*, and *LAMB2* variants and may occasionally result in Potter sequence, can help distinguish these forms from *NPHS1*- and *NPHS2*-related disease, in which glomerular filtration rate (GFR) is typically preserved at presentation. Nevertheless, the genotype–phenotype correlations are incompletely understood. Expanding access to genetic testing has refined these correlations, improved prognostic accuracy, informed long-term treatment strategies such as kidney transplantation, and enabled genetic counseling of the family. This review provides general information on the genotype–phenotype association in monogenic disease and summarizes available information on the phenotypes associated with the CNS and iNS variants.

## The principles of genotype–phenotype correlation in monogenic disease

**Fig. 1:**
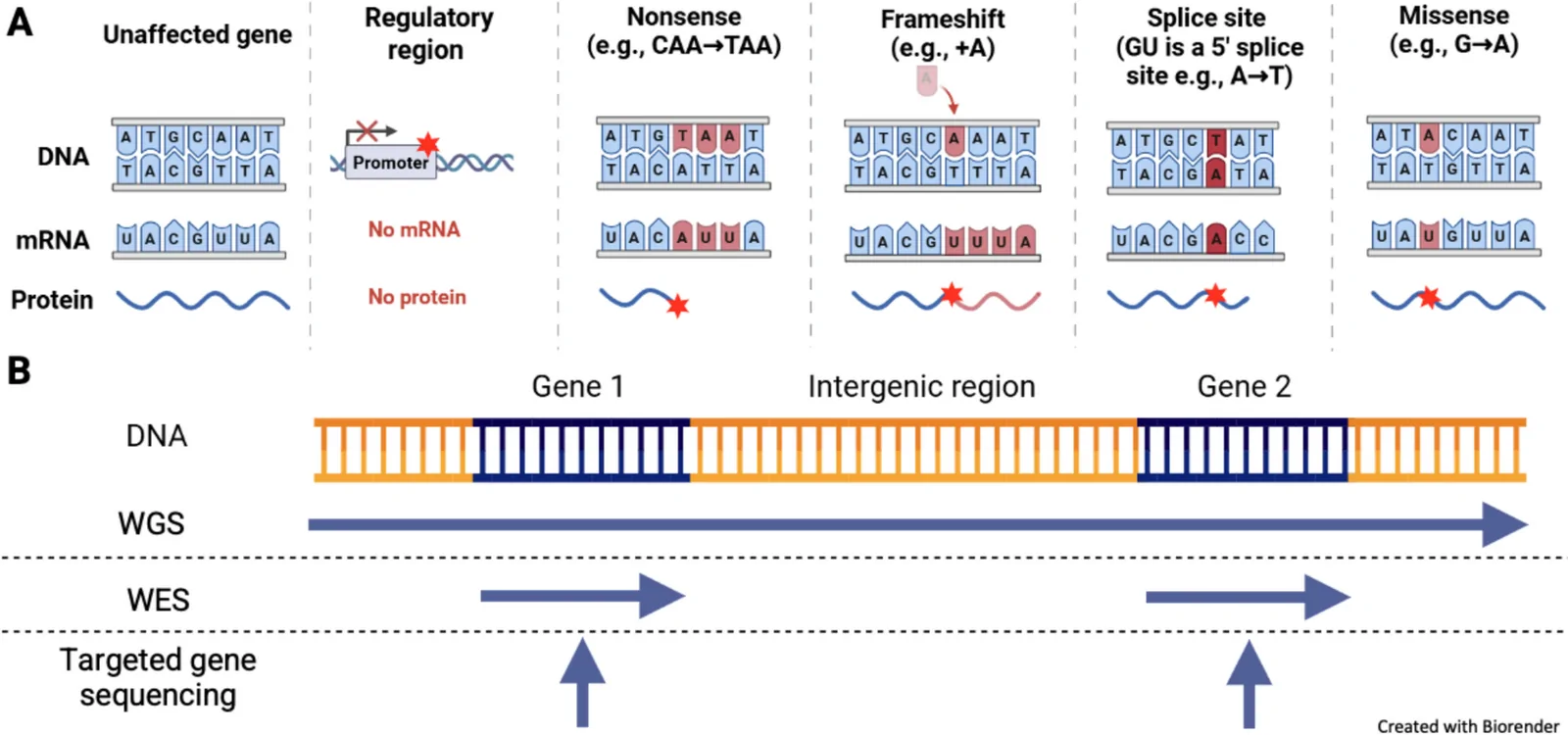
Classes of pathogenic variants and sequencing strategies used to detect them. (**A**) Schematic of how different variant classes disrupt the flow of genetic information from DNA to mRNA to protein. In an unaffected gene, DNA is transcribed into mRNA and translated into a functional protein (wavy line). A variant in a regulatory region, such as the promoter, prevents RNA polymerase binding and abolishes transcription, so that neither mRNA nor protein is produced. A nonsense variant introduces a premature termination codon (PTC; e.g., CAA → TAA), leading to a truncated protein or, more commonly, transcript degradation by nonsense-mediated decay. A frameshift variant, caused by an insertion or deletion of a number of bases not divisible by three (e.g., + A), shifts the reading frame and typically produces a PTC downstream, yielding a truncated or nonfunctional protein. A splice site variant alters a canonical donor (5′; e.g., A → T at a GU dinucleotide, as shown) or acceptor (3′) site and causes exon skipping, intron retention, or use of a cryptic splice site, resulting in an altered protein. A missense variant changes a single base pair (e.g., G → A) and substitutes one amino acid for another, which may impair protein folding, stability, or function. Red stars mark the nucleotide or protein positions affected by each variant. (**B**) Genomic coverage of the three sequencing strategies discussed in the text, illustrated on a segment of DNA containing two genes (dark blue, coding regions) separated by an intergenic region (orange). Whole-genome sequencing (WGS) interrogates the entire genome, including coding, non-coding, and intergenic regions. Whole-exome sequencing (WES) is restricted to protein-coding exons. Targeted gene sequencing (gene panels) examines a predefined set of genes selected for their relevance to the clinical phenotype. Created with Biorender

The clinical severity of monogenic disease is determined by the biological role of the affected gene, the type of variant affecting the gene, the extent of protein dysfunction, and the pathogenic effects of dysfunctional proteins. In general, loss-of-function variants (e.g., nonsense, splicing, or frameshift variants that abolish protein expression) are associated with severe, early-onset disease, while hypomorphic variants (e.g., missense variants that partially impair protein function) tend to result in milder or more variable phenotypes (Table 1, Fig. 1) [24, 28, 30]. Nonsense variants trigger a quality-control mechanism in affected cells, called nonsense mediated decay (NMD), which degrades faulty mRNA containing a premature stop codon (PTC) [31]. This process prevents the production of truncated, potentially harmful proteins and helps maintain normal gene expression. If NMD is bypassed, it may lead to accumulation of misfolded proteins toxic to affected cells. Currently, there are no published studies examining NMD in premature stop codon variants associated with NS.

**Table 1:** The impact of variant type on the protein structure and function

Variant type and definition	Consequences for protein structure and function
Regulatory: Variant in a non-coding regulatory element (promoter, enhancer, silencer, insulator, untranslated region) that affects transcription, mRNA stability, or translation efficiency	Reduced expression leading to haploinsufficiency; overexpression (potentially oncogenic, as with MYC or TERT promoter variants); loss of tissue-specific expression; aberrant developmental or circadian timing of expression; or imbalanced isoform expression through alternative promoters or polyadenylation sites
Nonsense: Single nt substitution that introduces a PTC in the mRNA	Truncated, typically nonfunctional protein, or no protein if the transcript undergoes nonsense-mediated decay. Specific consequences include loss of catalytic activity (missing domains), impaired localization or transport (truncated signal peptides), disrupted multimer assembly (potentially dominant-negative), and altered post-translational modifications or binding interfaces
In-frame deletion or insertion: Insertion or deletion of nts in a multiple of three, preserving the reading frame	Addition or removal of one or more aa. Consequences depend on the position, the physicochemical properties of the affected residues, and effects on protein stability and dynamics, and may include loss of function, partial activity, gain of function, altered substrate specificity, or dominant-negative effects
Frameshift: Insertion or deletion of nts in a number not divisible by three, shifting the reading frame	Altered downstream aa sequence, usually followed by a PTC. Produces a truncated or aberrant protein that is typically nonfunctional and, in some cases, toxic
Splice-site: Variant disrupting a canonical donor (5′) or acceptor (3′) splice site	Aberrant mRNA maturation through exon skipping, intron retention, or use of a cryptic splice site, which may introduce a frameshift and PTC. Typical protein-level consequences include truncation, loss of functional domains, misfolding, loss of function, and in some contexts dominant-negative or toxic gain-of-function effects
Missense: Single nt substitution that changes a codon and therefore the encoded aa	Single aa substitution, with consequences ranging from no effect, through partial loss of function, to gain of function, toxicity, or dominant-negative interference with the wild-type protein
Compound heterozygous genotype: Two different pathogenic variants in the same gene, one inherited from each parent, at different positions within the gene	Functional consequences depend on the nature and position of each variant, with reduced or absent protein function according to the severity and residual activity of each allele. Phenotypic variability among individuals sharing the same genotype may arise from epistasis, modifier genes, or environmental factors. Diagnostically challenging, as each variant considered alone may appear benign or be classified as a VUS

Abbreviations: *nt* nucleotide, *aa* amino acid, *MYC* myelocytomatosis, *TERT* telomerase reverse transcriptase, *PTC* premature termination codon, *VUS* variant of uncertain significance

A missense variant is a change in a single nucleotide that results in the substitution of one amino acid for another in the protein sequence. Depending on the location and properties of the change, it can either have no effect or disrupt protein function and cause disease. For example, *NPHS1* variants affecting the intracellular domain are associated with longer survival time than variants affecting the extracellular domain [32]. It is important to mention that some genes are associated with more severe phenotypes than others. For example, truncation and frameshift variants in *NPHS1* and *LAMB2* genes encoding essential slit diaphragm proteins present with the most severe phenotype leading to early-onset and rapid progression to kidney failure [14, 33]. On the other hand, missense variants differ in severity [7, 33–35]. Compound heterozygous missense variants in podocin contribute to later onset and delayed kidney failure [36]. Understanding these distinctions is critical for developing a personalized approach to diagnosis, prognosis, and management of early-onset monogenic NS.

## The genotype–phenotype correlations in *NPHS1* variants

The *NPHS1* gene, located on chromosome 19q13.1, comprises 29 exons encoding nephrin composed of an extracellular domain containing eight immunoglobulin-like (Ig) repeats and a fibronectin type III (FN-III) domain, followed by a single-pass transmembrane domain, and a cytoplasmic domain [7, 37–39]. Nephrin is expressed in kidney podocytes at the slit diaphragm between neighboring podocyte foot processes. This positioning enables nephrin to serve a crucial function preserving the integrity of the glomerular filtration barrier. Nephrin extracellular domain binds to the extracellular domain of nephrin from the neighboring podocyte foot processes, forming homodimers and to additional proteins, including ephrin-β1, forming heterodimers [40, 41]. These interactions promote barrier function, cellular adhesion and signaling, both inside-out and outside-in. Nephrin intracellular domain contains nine tyrosine residues that undergo phosphorylation [42]. Nephrin phosphorylation is essential for podocyte signaling, that regulates actin polymerization and dynamics that, in turn, regulate motility and survival, and preserves the structural and functional integrity of the glomerular filtration barrier [42–44].There have been reported 1031 benign or likely benign variants and 543 pathogenic or likely pathogenic *NPHS1* variants [45]. The pathogenic *NPHS1* variants disrupt podocyte function and are the most common causes of CNS worldwide [8, 11, 12, 14, 46]. In certain instances, a milder phenotype of *NPHS1* variants can result in steroid-resistant nephrotic syndrome (SRNS), which may manifest later in childhood or in adulthood [7, 15, 47].

### *NPHS1* nonsense and frameshift variants

The frameshift variant Fin-major (c.121delCT; p.L41fs*91) and the nonsense variant Fin-minor (c.3325C > T; p.R1109*) are responsible for the majority of CNS cases in Finland. Fin-major has a prevalence of 78% among the CNS cases in the Finnish population [7, 15]. It is caused by deletion of 2 bps in exon 2 of *NPHS1* gene, causing a frameshift that results in a short fragment truncated in the early extracellular domain, not detected in kidney biopsies [7, 24, 37, 48]. Fin-minor is a nonsense variant in exon 26 with a prevalence of 16% among CNS cases in Finland. The resulting nephrin fragment, comprising 1109 amino acids, is predicted to have intact extracellular and transmembrane domains and a severely truncated intracellular domain. Despite the prediction, immunofluorescent staining and western blot analysis of nephrectomy specimens from Fin-minor patients suggested absent glomerular nephrin expression [24, 48]. Children with these variants encounter significant morbidity resulting from massive protein loss and develop kidney failure requiring chronic kidney replacement therapy, and eventually kidney transplantation within the first 2–3 years of life [26, 27, 49]. Historically, the life expectancy of children with these variants without supportive treatment was only 7.6 months to 5 years [1, 13, 49, 50].

Another nonsense variant causing CNS is c.3478C > T, p.R1160* that lacks the last 81 amino acids of the cytoplasmic tail domain. Although usually associated with severe disease, this variant was linked to milder or variable phenotypes in Maltese, Bangladeshi, and Indian patients, with possible gender effects, with females sometimes exhibiting a milder course [8, 24, 51]. Specifically, two patients with homozygous p.R1160* nephrin had normal GFR at 10 years of age. Another study showed that three patients with severe proteinuria at birth, later had normal GFR at the ages of 6 months, 1.5 years and 2.5 years, respectively [52]. In contrast to Fin-minor (p.R1109*), glomerular nephrin expression was detected in patients homozygous for p.R1160* independent of disease severity [24]. The mechanisms for the difference in nephrin abundance between the two variants, pR1109* and p.R1160* as well as the variable clinical severity in individuals homozygous for p.R1160* remain unknown. Future mechanistic studies may provide answers for such variability. Predicting severity is critical to prognosticate and plan for the appropriate treatment plan, such as supportive care or management with nephrectomy and kidney transplantation. Mechanistic studies could also guide the development of variant-specific therapeutic strategies.

Another illustrative example is c.1481delC, p.K494fs*54, a founder variant highly enriched in the Old Order Mennonites of Lancaster County, Pennsylvania (United States), where the carrier frequency reaches approximately 8% and the incidence of CNS is ~ 1 per 500 live births, a roughly 20-fold increase over the Finnish baseline [53]. The deletion shifts the reading frame at codon 494 and introduces a PTC 54 codons downstream, producing a 547-residue truncated nephrin that lacks the transmembrane domain and is therefore predicted to be a null allele. The variant presents with a clinical picture of placental enlargement, low birth weight, and prematurity, and massive proteinuria soon after birth.

Of note, frameshift variants reported before approximately 2015 often follow an older nomenclature in which the number after "fs*" or "fsX" denotes the absolute protein position of the new stop codon, and the new amino acid introduced at the frameshift site is omitted (e.g., p.L41fs*91 for Fin-major). Current Human Genome Variation Society (HGVS) recommends the offset convention, p.(L41Dfs*50), in which the number after termination (*) refers to codons read in the shifted frame before the premature stop, and the new amino acid at the frameshift codon is explicitly given [54]. Throughout this review, frameshift variants are reported in their originally published form to preserve traceability to the source literature; the modern HGVS-compliant designation is given in parentheses in Table 2 where the two differ.

**Table 2:** Representative genotype–phenotype correlations for variants associated with CNS and iNS. Variant designations are given in the form originally published, followed in parentheses by the current Human Genome Variation Society (HGVS)-compliant nomenclature [54]

*NPHS1* (nephrin; 19q13.1)					
Variant	**Type**	**Mutant protein expression**	**Mutant protein function**	**Phenotype**	**Ref**
p.L41fs*91 (p.L41Dfs*50) Fin-major	Frameshift	Absent from the SD; no detectable full-length or truncated protein	Complete loss of function; absence of SD on electron microscopy	Classical severe Finnish-type CNS; accounts for ~ 78% of Finnish alleles	[7, 48]
p.K494fs*54 (p.K494Rfs*54) Mennonite founder	Frameshift	Predicted absent; truncated 547-aa fragment lacks transmembrane domain	Null allele	Severe CNS indistinguishable from Fin-major homozygotes	[53]
p.R1109* (Fin minor)	Nonsense	Absent in kidney tissue (equivalent to Fin-major)	Complete loss of function; SD absent	Classical severe CNS; ~ 16% of Finnish alleles	[7, 37, 48, 122]
p.R1160*	Nonsense	Detected both in vitro and in vivo	Truncated protein lacks the portion of cytoplasmic tail required for interaction with podocin; impaired SD signaling	Variable severity; reported in both classical CNS and milder late-onset disease	[24, 43, 122]
p.S350P	Missense	Expression level not characterized in published studies	Substitution in Ig-5; functional effect undetermined	Milder CNS	[67]
p.L587R	Missense	Likely ER retention with reduced cell surface abundance	Substitution in Ig-6; impaired function	Variable severity	[24, 35, 55, 123]
p.V709G	Missense	Likely ER retention; specific expression data not published for this variant	Substitution in the linker between Ig-6 and Ig-7; impaired function	Milder CNS	[12]
p.G715R	Missense	Likely ER retention; specific expression data not published for this variant	Substitution in the linker between Ig-6 and Ig-7; impaired function	Severe CNS	[35]
*NPHS2* (podocin; 1q25-q31)					
Variant	**Type**	**Mutant protein expression**	**Mutant protein function**	**Phenotype**	**Ref**
p.Q129*	Nonsense	No functional protein produced	Termination within PHB domain; null allele	Classical severe CNS	[83, 124]
p.R138Q	Missense	Protein is produced but retained in the ER; fails to reach plasma membrane	Substitution in PHB domain; unable to recruit nephrin to lipid rafts; effectively null	Classical severe CNS	[81, 125–127]
p.R229Q	Missense	Protein is secreted and reaches the plasma membrane normally	Substitution in PHB domain; binds nephrin with reduced affinity; hypomorphic; pathogenic only when in trans with a more severe allele	Milder, late-onset NS	[85, 87, 88, 125]
WT1 (Wilms tumor protein; 11p13)					
Variant	**Type**	**Mutant protein expression**	**Mutant protein function**	**Phenotype**	**Ref**
p.R390*	Nonsense	Truncated protein, if produced, lacks the DNA-binding ZFs	Termination within ZF-3; null allele causing haploinsufficiency	WT predisposition, potentially with accompanying glomerulopathy	[25, 128]
c.1432 + 4 C > T c.1432 + 5G > A	Splice site (intron 9 donor)	Total WT1 expression is preserved; the + KTS/− KTS isoform ratio is markedly shifted toward − KTS	Disrupts alternative splicing at the exon 9 donor; abolishes production of the + KTS isoform required for normal podocyte function	Frasier syndrome (FSGS with gonadal dysgenesis in 46,XY)	[129–131]
p.R394W	Missense	Mutant protein is produced at normal levels	Substitution in ZF-3 impairs DNA binding; exerts dominant-negative effect on wild-type allele (autosomal dominant inheritance)	Denys–Drash syndrome; FSGS with diffuse mesangial sclerosis	[132–134]
*LAMB2* (Laminin β−2; 3p21.31)					
Variant	**Type**	**Mutant protein expression**	**Mutant protein function**	**Phenotype**	**Ref**
p.Q125* p.R964*	Nonsense	Severely reduced or absent laminin β2 expression in the GBM	Truncation at LN domain (Q125) or mid-protein (R964); no functional protein incorporated into laminin-521	Classical severe Pierson syndrome (CNS, microcoria, neurodevelopmental involvement)	[33, 135]
p.L139P p.R246W	Missense	p.L139P: reduced secretion with impaired expression in the GBM p.R246W: markedly reduced protein accumulation in the GBM (~ sevenfold reduction)	Substitutions in the LN domain disrupt laminin polymerization at the polymerization node; p.R246W additionally causes impaired secretion	Classical severe Pierson syndrome	[33, 136]
p.H147R	Missense	Partial expression preserved; hypomorphic allele; some residual protein in the GBM	Substitution in the LN domain; impairs but does not abolish laminin polymerization	Milder Pierson variant	[137]
p.C321R	Missense	Protein misfolds and is retained in the ER; secretion to the GBM is reduced; induces podocyte ER stress; partial rescue in vitro with the chemical chaperone TUDCA	Substitution of conserved Cys in the Lea1 module disrupts a stabilizing disulfide bond; hypomorphic allele with some residual function	Less severe Pierson variant; CNS with mild extrarenal features	[138]
*PLCE1 *(Phospholipase C Epsilon 1; 10q23.33)					
Variant	**Type**	**Mutant protein expression**	**Mutant protein function**	**Phenotype**	**Ref**
p.R493* p.R1116* p.Q1616*	Nonsense	Truncated protein, if produced, lacks C-terminal catalytic and regulatory domains	Complete loss of enzymatic activity	Variable	[14]
p. S1484L	Missense	Protein expression preserved; specific secretion/stability data not published; hypomorphic allele	Substitution in the PLC-X catalytic domain; impairs but does not abolish catalytic activity; hypomorphic allele	Milder	[99]

Asterisk is used to denote mutation, either frameshof or premature termination. Abbreviations: *SD* slit diaphragm, *Ig* immunoglobulin-like repeat, *ER* endoplasmic reticulum, *PHB* prohibitin homology domain, *ZF* zinc finger domain, *KTS* lysine-threonine-serine tripeptide encoded at the exon 9–intron 9 boundary of *WT1*; FSGS, focal segmental glomerulosclerosis; *GBM* glomerular basement membrane, *LN* laminin N-terminal domain, *TUDCA* tauroursodeoxycholic acid, *LEa1* laminin-type EGF-like module, *PLC-X* catalytic X domain of phospholipase C

### *NPHS1* missense variants

Missense variants alter the nephrin amino acid sequence and most commonly cause loss of protein function through misfolding, instability, impaired protein-protein interactions, and defective trafficking [55–61]. Less frequently, missense variants in podocyte genes confer gain of protein function, for example by activating the unfolded protein response, promoting misfolded proteins aggregation or the endoplasmic reticulum (ER)-associated degradation (ERAD), or generating aberrant binding new protein partners, causing cellular toxicity [62]. Gain-of-function NS variants have been described in genes other than *NPHS1* [63, 64]. The *NPHS1* missense variants discussed below are predominantly loss-of-function changes affecting the extracellular Ig-like repeats.

Several *NPHS1* missense variants cause defective folding and trafficking that can be partially rescued in vitro by chemical chaperones. The p.W64S variant produces ER retention with defective biosynthetic processing [55, 58]; treatment with 4-phenylbutyric acid (4-PBA) restored trafficking, allowing W64S nephrin to colocalize with Neph1 at the plasma membrane and undergo phosphorylation by Src-family kinases. Variants p.I171N, p.G270C, p.S366R, p.S724C, and p.R743C, all in the Ig-like repeats, showed similar ER retention, reduced glycosylation, decreased plasma membrane abundance, and degradation through ERAD [55, 56, 58]. Of these, p.I171N, p.G270C, and p.S366R were rescued by 4-PBA [58]; castanospermine partially restored p.I171N and p.S366R surface expression to approximately 30–35% of wild-type levels [58]. The fact that these shared trafficking defects are reversible under chaperone treatment defines a class of variants for which proteostasis-directed therapy is a plausible long-term strategy.

In contrast, two missense variants illustrate how clinical severity can diverge from biochemical prediction. The homozygous p.L587R variant, located in the Ig-6 repeat, was associated in one sibling pair with partial remission characterized by persistent proteinuria but preserved serum albumin and no edema most likely reflecting the antiproteinuric effect of angiotensin converting enzyme (ACE) inhibition rather than immunosuppression from corticosteroids [11, 65]. The p.S350P variant was identified in a patient with a notably mild clinical course of NS diagnosed at 2.5 months but with normal creatinine and only non-nephrotic range proteinuria at 10.9 years [66]. Substitution of polar serine with non-polar proline would typically be predicted to disrupt secondary structure, since proline’s rigid cyclic side chain destabilizes α-helices or β-strands [67]. However, Ig-like domains consist of antiparallel β-sheets connected with loop regions [68]. Loop regions are known to tolerate and indeed enrich for proline residues [69]. The mild phenotype is therefore consistent with p.S350P falling within a loop rather than within the β-sheet core, where the same substitution would be expected to destabilize the domain.

At the severe end of the spectrum, the c.2143G > C, p.G715R variant caused early-onset disease at three months of age and progression to kidney failure by two years of age; the patient subsequently lost two kidney transplants due to rejection [35].

Taken together, these examples highlight two predictors of *NPHS1* missense severity: the position of the variant within a structural element, β-sheet core *versus* loop or conserved interaction surface *versus* periphery, and the magnitude of the physicochemical change, with substitutions between residues of similar polarity and charge tending to be milder than those that introduce charge or steric mismatches. Mechanistic dissection of these variants, particularly those rescuable by chemical chaperones, may guide development of variant-tailored proteostasis therapies aimed at restoring nephrin folding and slit diaphragm integrity.

Variant c.1170 + 2 T > C located in the intronic splice region of exon 9 was detected in two patients and they both died within three months of age [70]. The clinical severity of splice-site variants is particularly difficult to predict, as they can introduce multiple types of genetic alterations, including exon skipping, changes in exon number, or the creation or loss of splice donors, acceptors, and enhancer sites. Consequently, these variants are generally associated with severe phenotypes and poor clinical outcomes. Studying such variants remains challenging due to their complex molecular consequences, and therapeutic options for these cases are likely to rely on personalized gene-based strategies (Table 2) [71].

Philippe et al. demonstrated that compound heterozygous *NPHS1* variants can cause later-onset SRNS [60]. The variants included truncating, splice-site, and missense changes. Notably, most missense variants in that cohort trafficked normally to the plasma membrane and retained nephrin homodimerization capacity and Neph1 interaction, suggesting residual protein function. The presence of at least one “mild” allele with retained partial function likely explains the later-onset and milder clinical course in these patients. Conversely, large CNS cohort studies have found limited *NPHS1* genotype–phenotype predictive value in non-Finnish patients once CNS is established. Bérody et al. found no significant differences in age at diagnosis, kidney failure, death, or albumin withdrawal between “severe” and “mild” genotype groups [27]. Dufek et al. further showed comparable baseline parameters, similar response to ACE inhibitors, and comparable renal and patient survival across genotypic groups [26]. These findings collectively highlight the limited predictive value of *NPHS1* genotype severity once CNS is established, and underscore that individualized clinical monitoring remains essential regardless of genotype.

An important clinical consideration in *NPHS1*-associated CNS is the risk of de novo post-transplant glomerulopathy. Recurrent NS after transplantation has been documented in approximately 25% of patients homozygous for Fin-major, with a mean time to onset of 12 months [72]. This complication is considered immune-mediated, driven by anti-nephrin antibodies directed against nephrin as a neoantigen in the kidney allograft [73]. Affected patients may respond to plasma exchange and immunosuppression, as outlined in the ESPN-ERKNet management guidelines [28, 29]. However, the risk of allograft loss is high, occurring in approximately half of the affected cases [72]. Whether patients homozygous for Fin-minor carry a similar risk remains uncertain. The longer truncated nephrin fragment produced by Fin-minor may confer partial immune tolerance; however, nephrin protein was absent from the glomeruli of patients with biallelic Fin-minor variant, raising concern for a transplant risk comparable to that seen in Fin-major homozygotes.

## The genotype–phenotype correlations in *NPHS2* variants

Podocin is a lipid raft-associated protein localized at the glomerular foot process membrane, specifically at the insertion site of the slit diaphragm [74, 75]. It is encoded by the *NPHS2* gene, which is mapped to chromosome 1q25 [76]. Podocin contributes to the formation and stabilization of the slit diaphragm by interacting with proteins such as nephrin and CD2AP. *NPHS2* variants disrupting podocin expression or function present with NS of variable severity [47, 76–78]. Some variants manifest at birth, whereas others manifest later in infancy or early childhood, typically exhibiting a slower progression to kidney failure in comparison to *NPHS1* mutations [24, 47, 79, 80].

The class of variants provides important prognostic information regarding both age of onset and disease severity. The p.R138Q variant is the most frequent *NPHS2* pathogenic allele in European cohorts, accounting for approximately 32% of all *NPHS2* variants, and is typically associated with early-onset CNS [76]. Kidney biopsy may show focal segmental glomerulosclerosis (FSGS) or minimal change disease. Despite its early presentation, disease progression to kidney failure is generally slower than in *NPHS1*-related CNS. Functionally, p.R138Q is retained in the ER, consistent with a severe loss-of-function effect leading to earlier onset [8, 81].

In contrast, missense variants such as p.V180M and p.R238S are associated with later disease onset, consistent with functional studies showing that, unlike p.R138Q, these proteins variants can traffic to the plasma membrane [8, 81]. Conversely, truncating variants, such as p.R196*, are linked to severe, early-onset CNS, reflecting the profound loss of functional podocin [82]. Compared with *NPHS1*, *NPHS2-*associated CNS cases generally progress more slowly [8, 81].

A comprehensive mutation update by Bouchireb et al. identified 126 pathogenic *NPHS2* variants, including 53 missense, 17 nonsense, 26 small deletions, 11 insertions, and 16 splice-site changes distributed across the entire gene, and established several clinically important genotype–phenotype correlations [83]. Among patients with steroid-resistant CNS or iNS in European cohorts, at least one truncating variant or homozygous p.R138Q was identified in 94.1% of cases, underscoring the predominance of severe loss-of-function alleles in this age group [83]. The p.R168H variant similarly conferred early-onset, with 61% of affected patients developing SRNS before 6 months of age, whereas p.V180M was consistently associated with later diagnosis, as 20 of 22 reported patients with this variant were diagnosed after 8 years of age, a finding explained by the ability of p.V180M to traffic to the plasma membrane and retain partial function.

Although *NPHS2*-associated nephrotic syndrome typically follows an autosomal recessive inheritance pattern, apparent pseudo-dominant transmission has been reported in several families [84, 85]. This is explained in part by the relatively high population frequency of the p.R229Q variant, which reaches an allele frequency of approximately 3% in Europeans and 6% in Finnish Europeans [86, 87]. The high European carrier frequency allows otherwise unrelated carriers to appear in successive generations, mimicking dominant inheritance. Notably, p.R229Q exhibits mutation-dependent pathogenicity: it causes disease only when in trans with pathogenic *NPHS2* variants affecting the C-terminal domain encoded by exons 7–8 [88, 89]. When p.R229Q is paired with such a C-terminal variant, the resulting mutant podocin heterodimer cannot traffic normally to the slit diaphragm, causing disease; when paired with pathogenic variants in exons 1–6, disease does not result [88, 89]. This interaction-dependent mechanism has important implications for genetic counseling, as compound heterozygosity for p.R229Q and a second *NPHS2* variant does not invariably confer disease risk [90]. A patient who carries p.R229Q in trans with a pathogenic variant located in exon 7 or 8 is at high risk for developing NS, which may not manifest before adulthood and is characterized by slower progression to kidney failure compared with patients carrying biallelic pathogenic *NPHS2* mutations [83, 87].

Significant interethnic variability in the mutation spectrum has also been documented, with p.R138Q predominating in European populations (30–40% of detected alleles), p.R138* almost exclusively observed in Israeli Arab patients, and p.A284V enriched in South American and Spanish cohorts, reflecting population-specific founder effects [83]. Finally, the exceedingly low rate of post-transplant recurrence of SRNS in patients with biallelic *NPHS2* mutations, in contrast to more than 90% recurrence seen in immune-mediated forms with secondary steroid-resistance, further supports the structural rather than immunological basis of podocin-related disease and reinforces that these patients should not receive immunosuppressive therapy [73, 83, 91, 92].

## The genotype–phenotype correlations in other gene variants

The *Wilms tumor 1 (WT1)* gene is a transcription factor with a critical role in kidney development. *WT1* variants correlate with severe NS and developmental anomalies, presenting a significant risk of swift progression to kidney failure and an elevated likelihood of Wilms tumor [9, 60, 93, 94]. In *WT1*, missense substitutions affecting DNA-binding residues (exons 8–9) strongly associate with diffuse mesangial sclerosis (DMS), very early SRNS, and rapid progression, whereas truncating variants confer the highest Wilms tumor risk but typically a later SRNS onset, and intron 9 splice-site variants most often present with Frasier Syndrome (Table 2) [9, 95].

Importantly, *WT1*-associated disease represents a phenotypic continuum rather than a set of discrete, mutually exclusive syndromes. While various combinations of kidney and extrarenal findings associated with *WT1* pathogenic variants were historically designated as distinct syndromes (e.g., Denys–Drash syndrome, Frasier syndrome, WAGR syndrome), current expert consensus recognizes these as part of a continuous phenotypic spectrum, and the traditional syndromic designations are no longer considered clinically helpful [93, 96, 97]. Many patients present with a milder phenotype that cannot be readily classified into any of these historical categories. Incomplete penetrance and variable expressivity are common. The most frequent extrarenal features include disorders of sex development in most 46, XY individuals, Wilms tumor, and less commonly congenital anomalies of the kidney and urinary tract (CAKUT) and gonadoblastoma.

The *LAMB2* gene encodes the β−2 chain of laminin, a crucial protein component of the glomerular basement membrane (GBM). *LAMB2* is crucial for maintenance and function of podocytes. *LAMB2* contains the laminin N-terminal domain (LN), EGF-like domains, IV domains, coiled-coil domain and a β loop. Variants in *LAMB2* result in severe NS and systemic complications, typically associated with a poor prognosis due to rapid progression to kidney failure and considerable morbidity from related syndromic characteristics (Table 2) [33, 98, 99]. *LAMB2* pathogenic variants cause Pierson syndrome, characterized by CNS and ocular abnormalities. Diseased phenotype correlates with *LAMB2* variants where truncating variants are associated with early-onset CNS (median 0.3 vs. 2.0 months for other variants) and earlier progression to kidney failure (median 1.3 vs. 60.0 months), whereas non-truncating variants follow milder trajectories. While the LN domain is critical for basement membrane assembly, non-truncating variants within this domain do not statistically confer worse prognosis than variants outside the LN domain [100].

*PLCE1* encodes for phospholipase C epsilon 1, an enzyme that catalyzes the hydrolysis of phosphatidylinositol-4,5-bisphosphate to generate two second messengers: inositol 1,4,5-triphosphate and diacylglycerol [101, 102]. *PLCE1* is involved in signaling cascades that regulate podocyte shape, actin cytoskeleton dynamics, cell–cell communication, and responses to injury. *PLCE1* variants lead to variable severity; certain patients exhibit severe CNS at birth, whereas others experience a milder disease trajectory, with inconsistent progression to kidney failure [10]. Variants in the *PLCE1* gene are rare but have been associated with DMS and rapid progression to kidney failure in infants and have been implicated as a cause of iNS/DMS in non-Finnish cohorts, although typically only rarely and sometimes as single heterozygous variants of uncertain pathogenicity (Table 2) [8]. Although *PLCE1*-related disease follows an autosomal recessive inheritance pattern, incomplete penetrance has been reported, which may complicate genetic counseling [103, 104].

## Syndromic forms of early onset NS

Galloway–Mowat syndrome (GAMOS; OMIM #251,300) is a rare autosomal recessive disorder characterized by the combination of early-onset SRNS, primary microcephaly, brain malformations, and global developmental delay, caused by pathogenic variants in at least ten genes encoding subunits of the KEOPS tRNA-modifying complex (*OSGEP, TP53RK, TPRKB, LAGE3, GON7*) or other regulatory proteins (*WDR73, WDR4, NUP107, NUP133, YRDC*) [105, 106]. The renal phenotype is variable, ranging from mild proteinuria to severe SRNS presenting in the first months of life, with approximately half of affected individuals progressing to kidney failure and a reported mortality rate as high as 47% [107]. Emerging genotype–phenotype correlations indicate that *OSGEP* pathogenic variants are associated with earlier onset of NS and shorter life expectancy compared to *WDR73* variants, while homozygous non-truncating *WDR73* variants tend to confer a milder renal course with lower rates of proteinuria [107, 108]. No disease-modifying therapy is currently available, and management remains supportive; early genetic diagnosis is therefore essential for accurate prognostication, guidance of kidney replacement therapy planning, and prevention of recurrence through family counseling and prenatal diagnosis.

Primary coenzyme Q10 (CoQ10) deficiency is a rare mitochondrial disorder caused by biallelic pathogenic variants in genes encoding the CoQ10 biosynthetic pathway, most notably *COQ2, COQ6, PDSS2*, and *COQ8B*, and accounts for up to 5% of genetic SRNS cases, with podocytes being particularly vulnerable to CoQ10-related bioenergetic impairment and oxidative damage [12, 17, 109]. Unlike the vast majority of monogenic causes of NS, CoQ10 deficiency represents a potentially treatable condition: a large international cohort study of 116 treated patients demonstrated that oral CoQ10 supplementation was associated with a median 88% reduction in proteinuria at 12 months and significantly better kidney survival compared to matched untreated controls (5-year kidney failure-free survival 62% vs. 19%), with *COQ6*-related disease showing the highest rate of complete proteinuria remission [17]. Genotype–phenotype correlations are emerging, with *PDSS2* and *COQ2* variants more often associated with multisystem disease including encephalopathy, seizures, and cardiomyopathy, *COQ6* associated with hearing loss, while *COQ8B* (*ADCK4*) variants typically present as isolated nephropathy with later onset, and individuals with later-onset disease generally showing better response to supplementation [12]. Early genetic diagnosis is therefore essential, as prompt initiation of life-long CoQ10 supplementation can decelerate kidney disease progression, prevent extrarenal organ damage, and improve neurological outcomes, making CoQ10 deficiency one of the very few monogenic causes of NS amenable to disease-modifying therapy [17].

## Genetic testing in CNS and iNS

The genetic testing approaches include traditional Sanger sequencing and the next-generation sequencing (NGS) [110]. Sanger sequencing, developed in the 1970 s, was the gold standard for decades, but NGS, emerging in the 2000 s, has revolutionized genomics by enabling high-throughput, massively parallel sequencing. While Sanger sequencing is extremely accurate, its speed, scale and cost are inferior to NGS. The NGS approaches include gene panels, whole exome sequencing (WES), and whole genome sequencing (WGS). The 2024 study by Cheng et al. demonstrated the highest detectability of pathogenic variants in SRNS was by WGS, followed by WES, and gene panels [111]. WGS has higher yield as it can detect several types of variants that are typically missed by WES, including structural variants, deep intronic variants, tandem repeat expansions, and variants in regions with poor exome coverage [112]. The cost-efficiency fared in the opposite direction, supporting the current stepwise approach [111]. Consistent with the high yield of WGS, the study by Soliman et al. identified monogenic disease in 56% of families with early-onset SRNS [113]. This compares to 24% diagnostic yield of WES in children with SRNS in study by Zhang et al. [114]. It is important to note that the diagnostic yield of genetic testing in CNS and iNS is substantially higher than in general pediatric SRNS cohorts. Using targeted exon sequencing, Sadowski et al. identified a single-gene cause in 69.4% of families with CNS and approximately 49.7% of those with iNS, compared to 29.5% overall in a broad SRNS cohort [115]. Warejko et al. reported a comparable yield of ~ 48% in CNS using WES [116]. These data highlight that early-onset NS represents the highest-yield indication for genetic testing among all forms of SRNS.

Genetic testing by WES or expanded gene panel, has been recommended by the 2021 ERKNet and the ESPN consensus guideline as the first-line diagnostic test ordered already during the initial evaluation of every patient with CNS [29]. WES includes exome capture followed by NGS to sequence all the protein-coding regions (exons) of the genome, which represent about 1–2% of the genome but harbor ~ 85% of known disease-causing mutations. Advantages of WES are coverage of all, approximately 20,000, protein-coding genes, diagnosis of rare or genetically heterogeneous diseases, and flexible re-analysis is possible as new gene-disease links are discovered [117]. Targeted screening, either for individual genes most associated with CNS (*NPHS1, NPHS2, WT1, PLCE1, LAMB2)* or iNS (*COQ2, COQ6, PDSS2, SGPL1, TRPC6, INF2, ACTN4*) or gene panels (custom or commercial: SRNS, glomerulopathy) is also an excellent initial approach. The targeted screening performed on NGS platforms combined with hybrid capture or amplicon enrichment techniques allows high-depth, multiplexed sequencing with low error rates. Emerging practice includes reflex to WES if the targeted screening is negative. This stepwise approach provides higher coverage of disease-relevant exons, reduced incidental findings, faster turnaround for clinical decision-making, and is more cost-effective; however, serial genetic testing may also delay the diagnosis and will be less attractive as sequencing prices drop. As sequencing costs continue to decrease, WGS will likely become the preferred first-tier test for suspected genetic disorders, particularly in pediatric populations where early diagnosis impacts management [112]; and new modeling data is pointing to WGS as more cost-effective for children with suspected genetic disorders. In addition to increased diagnostic yield, WGS offers a significant advantage over targeted gene panels in discovering new disease-associated genes, as WGS interrogates the entire genome without requiring prior knowledge of which genes to include, enabling identification of variants in novel candidate genes and expansion of known phenotypes [118].

The choice of initial screening, either WES or gene panels, depends on the clinical availability of the method and can be informed by the clinical suspicion based on family history, ethnic background, and extra-renal symptoms concerning for syndromic NS. Independent of the initial method, genetic testing should always be performed as part of the initial diagnostic workup [28, 29, 119]. Establishing the genetic diagnosis early is essential for informing correct management, including screening for extrarenal manifestations, prognosis and family counseling. It may lead to the identification of genetic defects representing phenocopies of NS [28].

NGS approaches typically use peripheral blood as the gold-standard sample due to its consistency and DNA quality, but saliva and buccal swabs are viable alternatives for less invasive collection. The choice depends on the patient population (less invasive and convenient), clinical context, and resource availability. Resources for information about disease-causing mutations and genetic testing include public databases (GeneReviews/NCBI Gene Tests/GTR, ClinVar/LOVD/HGMD), patient advocacy (National Organization for Rare Disorders, NORD), research registries (PodoNet), American Kidney Fund, and specialized commercial laboratories.

Genetic counseling services play a critical role in the management of CNS/iNS by facilitating informed decision-making through pretest counseling, discussing secondary findings when WES or WGS is performed, and providing comprehensive reproductive risk assessment for families. Pretest genetic counseling should address the rationale for testing, potential outcomes, and implications for management. When WES or WGS is performed, genetic counselors must discuss the possibility of identifying American College of Medical Genetics and Genomics (ACMG) secondary findings, medically actionable incidental findings unrelated to the primary indication for testing [120]. Post-test genetic counseling in CNS/iNS encompasses interpretation of genetic findings, translation of results into treatment and prognostic implications, coordination of family screening, and comprehensive reproductive risk assessment. Recurrence risk estimates depend on inheritance pattern and specific variants. For autosomal recessive forms (*NPHS1, NPHS2*), parents who are both carriers have a 25% recurrence risk with each pregnancy. For autosomal dominant forms (*WT1*), many cases are de novo, and affected parents have a 50% risk of transmitting the variant, though penetrance and expressivity vary by gene and specific variant. Additionally, genetic counselors explain the significance of identified variants and their genotype–phenotype correlations, providing critical extension of physician capabilities.

Negative result on a targeted gene panel or WES does not exclude a genetic etiology, since current short-read technologies have limitations in detecting structural and deep-intronic variants, repeat expansions, and variants in complex genomic regions. In coming years “long read sequencing” will replace short-read WES and WGS. Long-read sequencing generates DNA reads tens to hundreds of kilobases in length compared to the 100–300 base pairs of current commercial technology (short-read technologies) enabling detection of structural variants, repeat expansions, deep-intronic mutations, and variants in complex genomic regions that are frequently missed by standard WES and WGS. There is limited published data specifically examining long-read sequencing technologies in NS, though the broader rare-disease literature demonstrates that long-read sequencing can provide an additional diagnostic yield of 7–17% following negative short-read genome sequencing [121].

Variants of uncertain significance (VUS) identified during genetic testing should be reinterpreted periodically as new evidence emerges, in line with the ACMG variant classification guidelines. A VUS designation typically reflects limited published data at the time of testing rather than established biological uncertainty, and a substantial proportion are reclassified as pathogenic or benign as evidence accumulates. Large-scale genomic studies in underrepresented populations, particularly consanguineous communities in which rare recessive variants are enriched and more readily characterized, are expected to expand the genetic landscape of early-onset NS and accelerate the reclassification of currently unresolved variants [113]. Families should therefore be informed that a VUS classification is provisional and that periodic reanalysis is recommended, especially as population-level data and functional evidence continue to accumulate.

## Practice points for clinicians

The following practice points summarize the clinical implications of the genotype–phenotype data reviewed above.

(i) Test early. Genetic testing is indicated in all children presenting with NS in the first year of life. Monogenic CNS and iNS do not respond to immunosuppression, and early confirmation prevents exposure to ineffective and potentially toxic therapy.
(ii) Individualize surgical and transplant planning. The timing of nephrectomy, dialysis, and transplantation should be guided by growth, nutritional status, and genotype. Homozygotes for the *NPHS1* Fin-major variant carry an approximately 25% risk of de novo post-transplant glomerulopathy from anti-nephrin antibodies; this should be addressed in pre-transplant counseling, and plasma exchange with intensified immunosuppression is the standard treatment if it occurs. Evidence is insufficient to quantify the risk in Fin-minor homozygotes or other non-Finnish truncating genotypes that abolish nephrin expression, and these patients should receive equivalent counseling and post-transplant proteinuria surveillance.
(iii) Manage WT1 disease as a multisystem condition. Wilms tumor surveillance by kidney ultrasound every 3 months until age 7 years, karyotyping in all phenotypic females, and endocrinology referral for 46,XY individuals to evaluate disorders of sex development.
(iv) Counsel families on inheritance and reproductive options. Recurrence risk should be communicated according to inheritance pattern, and prenatal diagnosis and preimplantation genetic testing should be offered where applicable.
(v) Reanalyze variants of uncertain significance. VUS identified on gene panels or WES require periodic reinterpretation as new evidence accumulates. Families should be informed that classification may change over time and that reanalysis is recommended when new publications or population-level data become available.

## From genotype to prognosis: outstanding questions and priorities for personalized care

Genetic testing has become a cornerstone in the diagnosis and management of CNS and iNS, enabling prognostic stratification, guiding therapeutic decisions, including the avoidance of immunosuppression in monogenic cases, and informing the timing of nephrectomy and kidney transplantation.

Genotype-phenotype correlations, however, remain incompletely understood. In non-Finnish populations, the clinical course of *NPHS1*-related CNS is highly variable regardless of the specific variant, as demonstrated by large cohort studies and reflected in the ESPN-ERKNet guidelines [26, 27, 29]. It is still unknown, for example, why p.R1160* can produce markedly different disease trajectories among individuals, ranging from early-lethal CNS to a milder course that improves with age. Although frameshift and nonsense variants are generally predicted to cause severe disease through NMD or the production of truncated nonfunctional protein, exceptions suggest that position-dependent NMD efficiency and alternative splicing modify outcomes in ways that are not yet fully characterized. Interpretation is further complicated by the rarity of these disorders: small patient numbers make it difficult to distinguish true genotype-driven variability from differences in the timing and quality of clinical care. Post-transplant de novo anti-nephrin glomerulopathy illustrates this limitation. The risk is estimated at approximately 25% in Fin-major homozygotes, but reliable estimates are lacking for homozygous Fin-minor and other non-Finnish truncating genotypes because transplanted cohorts in each subgroup are too small to support firm conclusions. Closing these gaps will require mechanistic studies of variant-specific effects on protein folding, trafficking, slit diaphragm assembly, and podocyte signaling, paired with longitudinal, multicenter cohorts capable of resolving genotype-specific trajectories.

Each genetically characterized case simultaneously adds to a growing global repository of pathogenic variants that can inform the design of variant-tailored therapies, including CRISPR/Cas9 genome editing and antisense oligonucleotide strategies aimed at protein restoration or stabilization. Embedding comprehensive genetic testing into routine clinical practice, particularly in neonates and infants, where early genomic information can prevent premature or unnecessary interventions, including transplantation, will be essential to realize truly personalized, disease-modifying care in CNS and iNS [71].

## Key summary points

1. Different variant types cause different disease severity.
2. Gene panels and WES remain practical first steps.
3. Early genetic testing improves care.

## Multiple choice questions

Answers are given following the reference list.

1. Which variant type is most likely to produce a completely aberrant and nonfunctional protein due to disruption of the reading frame?
  A. Missense
  B. Frameshift
  C. In-frame deletion
  D. Promoter disruption
2. Compound heterozygous missense variants in *NPHS2* (podocin) are often associated with later-onset nephrotic syndrome because:
  A. Both variants typically activate nonsense-mediated decay.
  B. Podocin is not required for slit diaphragm integrity.
  C. Missense variants always result in complete loss of protein expression.
  D. Missense variants usually preserve partial residual protein function.
3. Why do truncating variants in *NPHS1* or *LAMB2* typically cause severe, early-onset nephrotic syndrome?
  A. Both genes encode proteins that are dispensable for glomerular filtration.
  B. The truncated proteins acquire toxic gain-of-function activity.
  C. The loss of essential slit diaphragm components impairs filtration barrier formation shortly after birth.
  D. The truncating variants increase mRNA stability and protein abundance.
4. What is the main downside of doing a gene panel first and WES only if it is negative?
  A. It reduces accuracy.
  B. It increases incidental findings.
  C. It may delay the diagnosis.
  D. It cannot detect *NPHS1* variants.
5. Why is early genetic testing important in CNS and iNS?
  A. It helps confirm monogenic disease, guide prognosis, and avoid unnecessary treatments
  B. It determines which immunosuppression to start.
  C. It replaces the need for a kidney biopsy in all cases.
  D. It removes the need for family counseling.

Answers: B; 1. D; 2. C; 3. C; 4. A; 5.

## Supplementary Information

Below is the link to the electronic supplementary material.

Graphical abstract (PPTX 385 KB)
